# Special issue “reflecting on Freire: a praxis of radical love and critical hope for science education”—*theme: transnational collaborations and solidarities*: Transnational conversations about science education: Paulo Freire, interculturality and socio-political transformation

**DOI:** 10.1007/s11422-023-10163-6

**Published:** 2023-03-04

**Authors:** Haira Gandolfi

**Affiliations:** Faculty of Education, University of Cambridge, Cambridge, UK

**Keywords:** Interculturality and Decoloniality, Science education, Socio-political transformation, Teacher education

## Abstract

In this article I will examine some of the issues raised by the following three articles in this special issue about Paulo Freire and science education: Jenny Tilsen’s “The freshness of irreverence”: learning from ACT UP towards socio-political action in science education”; Suzani Cassiani and Irlan von Linsingen’s “Freirean inspirations in solidary internationalism between East Timor and Brazil in science education”; and Gonzalo Peñalosa, Jairo Robles-Piñeros and Geilsa Costa Santos Baptista’s “Science Education and Cultural Diversity: Freire’s Concept of Dialogue as Theoretical Lens to Study the Classroom Discourse of Science Teachers”. Brought together within this special issue under the theme of *Transnational collaborations and solidarities*, these articles explore the possibilities and tensions that emerge from thinking and practicing a Freirean-inspired science education that enables socio-political action and transformation by marginalised communities across the world. In this review, I will focus on ideas raised (to different extents) across these articles around three interrelated areas—interculturality and decoloniality, socio-political transformation, and teacher education and work—with the aim of expanding on what transnational inspirations and collaborations such as the ones promoted by this special issue can mean to those of us across the world working against the grain of marginalisation and dehumanisation (of students and teachers) from within science education.

In 2021 we celebrated the centenary of Paulo Freire (1921–1997), a thinker and practitioner of education who was not only influential in his country of origin, Brazil, but who also went against the traditional gaps of knowledge production and dissemination between Global North and Global South countries to authoring one of the most cited books in the area of social sciences in the world (https://blogs.lse.ac.uk/impactofsocialsciences/2016/05/12/what-are-the-most-cited-publications-in-the-social-sciences-according-to-google-scholar/): the seminal *Pedagogy of the Oppressed*, first published in 1968. As a result, Freire’s legacies to educational thinking and practices around the notions of critical consciousness, dialogic communication, praxis, socio-political action, emancipation and, more generally, social justice in and through education can be found in the works of several other prominent thinkers and activists across the world, such as Bell Hooks (1993), Steve Biko (see Khoapa 2008), Henry Giroux (2010) and Peter McLaren (1999).

And while Freire’s works have been often seen as linked to challenging social class-based injustices (Valladares 2021), they have also been used and expanded to ground reflections on educational issues that intersect with other axis of power, such as gender (sexism) and culture (racism and xenophobia), as argued, for instance, by Abdeljalil Akkari and Peri Mesquida (2008) in their Freirean work around *education and silenced voices*. As illustrated by different scholarship from across the world, including several articles in this special issue, Paulo Freire’s work has then the potential to be *transnational*: it can support decolonial and anti-colonial initiatives in different Global South communities with diverse histories of colonisation and intersectional oppressions (e.g. Akkari and Mesquida 2008; Khoapa 2008; Tarlau 2015; Peñaloza et al. and Cassiani and von Linsingen, in this special issue); anti-segregation and Black emancipation (e.g. Hooks 1993), and LGBQT + initiatives (e.g. Tilsen in this special issue) in the Global North; and networks of solidarity among radical teachers, such as the groups from the USA to Palestine described in the book edited by John Mink (2019). This is not to say that Freire aimed for his work to have a “universalist” nature to be applied in a similar way to any context of oppression across the world:

For example, it is a mistake—and when I say a mistake I am being polite—for a nation, a state, to think that it can educate other societies and other peoples. It is as if, for example, Brazil, impregnated with power (and fortunately this does not exist), decided to educate the world through Paulo Freire. Then Brazil would send Freire to Asia, Africa, North America, to teach other peoples to be like Brazilians. This would be absurd, this is absurd, and the name for this is imperialism. Besides this political dimension, we have also, a philosophical mistake in this, a cultural mistake, a misunderstanding of what is the meaning of culture. I am a Brazilian, I am my language, I am my food, I am my weather, as you are your language, your weather, your food, your feelings, your dreams. And we cannot export dreams. Once, at the beginning of my travels around the world, I was asked, I don’t remember where, “Paulo, what can we do in order to follow you?” And I said, if you follow me, you destroy me. The best way for you to understand me is to reinvent me and not to try to become adapted to me. Experience cannot be exported, it can only be reinvented (Freire, Freire, de Oliveira and Giroux 2014, p. 17).

Here it is then important to recognise that some of Freire’s original ideas, developed essentially within working class and rural communities in Brazil, have been employed in other contexts without the essential care for context-specific sensitivity—see more also in Liliana Valladares’s (2021) recent article on the uses of his notion of *emancipation* in science education. And, as he alludes to in his critical quote above, this is in direct contradiction to Freire’s own views around the socioculturally contingent nature of the praxis of education. Nevertheless, it is also important to recognise that what Freire was keen to propose, especially in his later works in the 1990s after a period living in exile during the Brazilian military dictatorship (de Oliveira 2011), was his strong belief in intercultural learning (Freire, 1992): that is, in different groups and people coming together to learn from each other and to “share the world” in solidarity (Freire, de Oliveira and Giroux 2014).

And it is in this Freirean spirit of transnational solidarity that I will explore here some ideas put forward by other three articles published in this special issue: Jenny Tilsen’s *‘The freshness of irreverence:’ learning from ACT UP towards sociopolitical action in science education*; Suzani Cassiani and Irlan von Linsingen’s *Freirean inspirations in solidary internationalism between East Timor and Brazil in science education*; and Gonzalo Peñaloza, Jairo Robles-Piñeros and Geilsa Costa Santos Baptista’s *Science Education and Cultural Diversity: Freire’s Concept of Dialogue as Theoretical Lens to Study the Classroom Discourse of Science Teachers.* Having been born, raised and worked as science teacher in Brazil, and being now a teacher educator in an English university, Freire’s ideas have a legacy not only to my previous professional life in the Global South, but also to how I currently find myself educating teachers in a deeply multicultural and unequal society in the Global North. So the general question I pose here, one that I keep reflecting on as part of my work as an educator, is what can we learn from each other—as science educators—about the relevance of Freire’s ideas to the contemporary conceptualisation and practice of science education? To ground this transnational reflection, I will focus here on three themes that seem to emerge, to different extents, from Peñaloza et al.’s, Cassiani and von Linsingen’s, and Tilsen’s articles: interculturality; socio-political transformation; and teacher education.

## Interculturality and science education: Paulo Freire and decolonial perspectives

Only the colonizers have a culture, an art or a language, and are civilized citizens of the ‘saviour world’. The oppressed are said to have had no history before the merciful effort made by the colonizers; they are seen as illiterate, barbarian natives. Freire’s work in Guinea-Bissau aimed to destabilize the colonial fossilization of minds.

(Akkari and Mesquida 2008, p. 334)

In their article *Science Education and Cultural Diversity*, Peñaloza et al. walk us through important ideas around cultural diversity, interculturality and science education, an area of great prominence in this journal and that bears close links to the field of Multicultural Science Education in the Global North. As Paulo Freire had discussed since his first publications decades ago, cultural identities and histories ground the educational experiences of both students and teachers. As such, for those of us working on issues of cultural diversity and schooling, uses of education as a practice of homogenisation of those identities and histories, and of imposition of particular knowledge systems (epistemologies) and ways of being (ontologies), should be challenged. In other words, we need to confront the historical and contemporary uses of educational ideas, practices and structures—such as by (neo)colonial projects—to physically control (Woolford and Gacek 2016), epistemologically oppress (de Sousa Santos 2018), and racialise (Grosfoguel, 2004) particular communities, if we aim to destabilise the “colonial fossilization of minds”, in Akkari and Mesquida’s (2008) words above, and build new approaches to education that foster the humanisation and emancipation of diverse communities.

Within the specific case of science education, this recognition of cultural diversity generally involves the acknowledgment of the heterogeneity of knowledges and practices that emerge from diverse ways of inquiring into the natural world, going beyond those of what is considered to be *mainstream science*—i.e. what is produced by official scientific communities. From the tradition of *cultural pluralism* within postmodern studies, as reviewed by Deborah Pomeroy (1994) and Eva Krugly-Smolska (2013), then comes the importance of recognising, in the practice of science education, the knowledges, values and ways of living in the natural worlds of, for instance, peasant and indigenous communities. Grounded on this *recognition* approach within science education, Peñaloza et al.’s article in this special issue explores some seminal works, such as by Glen Aikenhead (1996) and William W. Cobern (1994, 1996), that aimed at reflecting on possibilities and obstacles to the interaction between the so-called mainstream scientific knowledge about the natural world and other knowledge systems often deemed to be *non-scientific*.

Nevertheless, as also argued by Peñaloza et al., in this scenario of exploring cultural diversity in science education it is also important to consider the limits of cultural inclusion strategies that are based solely on recognising the coexistence of different cultures, societies and communities in a diverse society—or, as these authors call, the limits of the *institutionalization of cultural diversity*. As argued by some scholars in the context of Global North education (e.g. Hickling-Hudson and McMeniman 1993; Begum and Saini 2019), there have been several approaches to cultural inclusion in education that focus on *add-on* and tokenistic strategies to address this issue of *recognition*, such as diversifying examples used in the classroom or dedicating days or months to the celebration of diverse communities (e.g. Black History Month). We can think about these approaches to *recognition* as a “cosmetic cultural diversification” strategy that operates within the imperatives of performativity: that is, exploring examples of *traditional, non-scientific* knowledge practices of marginalised communities (e.g. local herbal medicines), but then moving onto what is framed to be the *real* science that needs to be learned for official exams (Pomeroy 1994; Michie 2002).

So here I would like to briefly expand on Peñaloza et al.’s work on intercultural science education to go beyond “recogn[ising] the multiple facets that may exist within the territory of which the subjects are part” and “develop[ing] a science education sensitive to cultural diversity”, towards a *critical* perspective of intercultural science education that embraces an important facet of the *inter*-culturality that had great importance in Freire’s work: importance in Freire’s work: the facets of relations and complexity, as also explored by Cassiani and von Linsingen in this special issue. With that mind, in addition to the *recognition of diversity*, I believe that science education needs to engage with the deconstruction of a traditional dichotomic approach to knowledge systems often found in our field—i.e. scientific versus non-scientific; scientific versus traditional—and acknowledge the holistic and complex scenario in which diverse types of knowledge systems, including mainstream science, emerge, as also argued by Lyn Carter (2004; 2017). Therefore, beyond the notion of *epistemological pluralism* proposed by Peñaloza et al., I argue that another concept developed by Boaventura de Sousa Santos (2007) can further our work in establishing a critical intercultural work within science education: *ecologies of knowledge*, which “is founded on the idea that knowledge is interknowledge” (de Sousa Santos, 2007, p. 66), recognising that all kinds of knowledge are established through dynamic relationships among diverse perspectives and inputs, including mainstream—or “Western”, “modern”—science.

Within this frame of thinking based on ecologies of knowledge, more than recognising the existence of so-called scientific and non-scientific knowledge systems in our work as science educators, we need to actually recognise how this mainstream scientific knowledge that is being positioned as *different* to non-scientific knowledges has actually been, throughout the history of humankind and until today, intrinsically and dynamically intertwined with those very same knowledge systems that have been *othered* by oppressive socio-historical processes, such as colonial projects. As recent scholarship in the field of Science and Technology Studies (STS) have shown (Harding, 2008; Patiniotis, 2013)—and as argued by Cassiani and von Linsingen in this special issue when discussing Ramón Grosfoguel’s (2004) decolonial take on the geopolitics of scientific development—those scientific knowledge and practices have themselves widely diverse and complex sociocultural roots, since “[modern] science was itself built upon a global repertoire of wisdom, information, and living and material specimens collected from various corners of the colonial world” (Roy 2018).

Thus, from this decolonial perspective proposed by Grosfoguel (2004), a more critical intercultural science education can emerge: one that understands that what we often call *modern science*—the one usually found in most science curricula and framed in opposition to *non-scientific knowledge—*is a result of several exchanges and forced appropriations between different communities, and of the circulation of diverse types of knowledges and material resources promoted by complex, of often oppressive, historical and geopolitical processes, such as the colonial projects explored by Carter (2017) and myself elsewhere (Gandolfi 2021a). Within this perspective, we would then look at knowledge systems within a science lesson not simply as *plural* or *diverse*, but through a more holistic and non-fragmented—and less *othering*—approach to the sociocultural complexity around these systems, making visible the often-unequal relationships established between them, such as in the case of extraction of natural resources (minerals, plants, animals, etc.) and *traditional* knowledge from colonised communities to inform and foster *mainstream scientific* development.

And this is where I believe that Freire’s own perspective around *knowledge* (and the learning of it) can greatly support a more critical and decolonial take on cultural diversity within science education: *knowledge* as the development of critical reading of reality. To Freire, education should not be about subjects being able to develop superficial, fragmented knowledge of their reality at a surface level; that is, to survey the world and recognise *diverse* ways of knowing that world. It should actually be about being able to critically *deconstruct* the complexities of that world and then rebuild it through a less dichotomic and fragmented view. In other words, according to Freire, if we want our societies—including ourselves and our students—to be capable of overcoming sociocultural, economic, political, gender and racial forms of oppressions, we need practices around cultural diversity, including in science education, to go beyond simply recognising different ways of “reading the world”, in Freirean terms, and promote an in-depth understanding of the norms, values and socio-political interests at play behind knowledge systems, including the scientific ones (Carter 2004, 2017). This Freirean lens around cultural diversity in science education then brings forward an intrinsic decolonial approach to the area: one that actively deconstructs the links between knowledges systems and power structures within scientific endeavours, as illustrated by Cassiani and von Linsingen in their comment about eugenics and the marginalisation of diverse communities.

As I have already argued elsewhere (Gandolfi 2019; 2021a, b), this decolonial reading of the world then asks for both the recognition of cultural diversity in science education and the sociocultural and historical deconstruction of scientific practices as a key aspect behind understanding the diverse nature of scientific development itself, or the “nature of science” (NOS) in Peñaloza et al. This perspective is also central to Freire’s view on the potential of education for emancipation, when human agency grounded on a critical reading of our realities—e.g. a non-fragmented understanding of the intricate links between scientific and non-scientific knowledge—translates into action against unjust processes and scenarios. And contrary to Valladares (2021, p. 581), who recently argued that Freire’s notion of emancipation has been used by some in science education to legitimate a discourse of “science as a liberating and emancipating force that frees humans from local beliefs, myths, and ideologies in contexts where different forms of knowledge coexist (personal, popular, indigenous, traditional, rural, and mainstream academic knowledge) carr[ying] the risk of reinforcing a scientism and a neocolonialism”, I propose here that a truly Freirean work in science education should not aim at simply *recognising diversity in knowledge systems* or *bridging the gaps* between different cultural experiences, but also at *recognising intersectional structures of oppression* (e.g. racism, sexism, homophobia, etc.) that have been operating in our diverse societies, including in how scientific developments and practices happen and are lived by different communities.

Therefore, and as summarised by Cassiani and von Linsingen, emancipatory education, for Freire, is one that empowers all of those involved to not only critically understand—*read or denounce*—injustices in culturally diverse world, but also to reconstruct—*transform or announce*—it. As a result, for this Freirean process of transformation to happen so that science education can help challenge oppressions of particular cultures and communities instead of reinforcing them (Valladares 2021), this same science education needs to be positioned beyond *recognition* approaches to inclusive practices around cultural diversity: it needs to also be firmly positioned as a vehicle for socio-political understanding and transformation, via diverse knowledge systems, as I will further explore in the next section, and as also advocated by Tilsen in this special issue.

## Science education for socio-political transformation

In the previous section I argued that an important outcome of bringing a Freirean perspective into our work on cultural diversity in science education is that it is asks us to engage with an in-depth deconstruction of structures of power and oppression that keep certain knowledges and communities marginalised in relation to others. In this sense, Freire’s work can be considered central to those interested in how issues of decolonisation can be addressed within the field of education, as proposed by Akkari and Mesquida (2008) and also by Antonia Darder (2015). This is because Freire was interested in a kind of education that does not simply describe the world to students in a fragmented way, but that actually helps them to critically understand the complexities of that world through the development of critical consciousness about historical, sociocultural, economic and political aspects around the diverse knowledges they engage with throughout their lives. Freire’s ideas can then be intrinsically linked and central to decolonisation of education since this area also aims, among other things, at fostering a reflexive understanding and deconstruction of socio-historical legacies resulting from colonialisms and other unequal relationships to the production and teaching of *knowledge*.

And it is exactly in this decolonial landscape inspired by Freire that I believe science education can find its wider transformative potential for historically marginalised communities: one that not only recognises diverse forms of knowledges, but that also recognises and deconstructs mainstream, modern science’s own socio-political and normative affiliations (Carter 2004, 2017). For instance, Peter McLaren argued in an interview with Angela Calabrese Barton that the relationship between capitalism, power and production of scientific knowledge has deeply influenced science education: “the marriages between capitalism and education and capitalism and science have created a foundation for science education that emphasises corporate values at the expense of social justice and human dignity” (Barton 2001, p. 847). Therefore, contrary to its potential to promote emancipation and social justice for marginalised communities—as asked for by several articles in this special issue—essentially utilitarian, neoliberal and triumphalist views about science are advanced by school science without any critical reflection or acknowledgement of its complexities, including political, economic and ethical commitments. According to Freire (1972), this lack of engagement with the complex socio-political aspects embedded within scientific practices would then allow students and teachers access to only a “fragmented view” of how this knowledge system works, which in turn does very little in terms of supporting students and teachers to be actual actors and transformers of that system.

This Freirean perspective on what it takes for (science) education to be truly emancipatory—through critical consciousness and social transformation—brings us to the *socio-political turn* addressed by Tilsen’s article in this special issue. Intrinsic to a Freirean decolonial project for science education that moves the issues from simply *recognising* cultural diversity to *deconstructing and transforming* science education against sociocultural homogenisation and oppression, this socio-political turn encourages both teachers and students to explicitly discuss the historical and contemporary socio-political aspects within scientific endeavours—including its complex links to other knowledge systems—and take actions to counter uses of science to control and marginalise certain communities, a concern recently raised by Sara Tolbert and Jesse Bazzul (2017) and Valladares (2021). Despite triumphalist views of science claiming its importance to bettering the lives of all across the world, what historical and contemporary cases investigated by the STS field show us is a deeply uneven access to, and sometimes oppressive legacy of, these promised outcomes and benefits from scientific developments. One does not need to look far to identify examples: eugenics; environmental injustice and metal exploitation in Global South mines; the Tuskegee experiment; access to coronavirus vaccination; and the handling of medical research during the HIV/AIDS epidemic in the 1980s, as explored by Tilsen’s article.

This socio-political turn then prompts us to rethink what we mean when we ask science education to engage with notions such as “science and society”, “science and citizenship”, “scientific literacy” and “socio-scientific issues”. While unpacking these notions is beyond the scope of this article and has already been done by others (e.g. Bencze 2017; Carter 2004, 2017; Levinson 2018; Santos 2009; Schenkel and Calabrese Barton 2020; Valladares, 2021), what I would like to highlight here is that despite attempts to bring the links between science and society more systematically to the practice of education—or to *humanise* science—, most strategies still operate within an instrumental perspective that focuses on preparing students to be in a society that is simply given to them, as also argued by Wildson Santos (2009).

In other words, even more humanistic ideas about the links between science education and society still tend to focus on learning how to *live in* a diverse society informed by scientific knowledge—e.g. deciding which mode of transportation to use given current concerns about climate change—but not on how to actively *transform* that society into a more socially just one for all (Valladares 2021). As I argued elsewhere (Gandolfi 2022) together with Emily Eaton and Nick Day (2020), Lynda Dunlop, Lucy Atkinson, Joshua Edward Stubbs and Maria Turkenburg-van Diepen (2021), and Stuart Tannock (2020), that is the case, for instance, of most approaches to environmental education in the Global North, which tend to focus on people’s (e.g. students) own individual subjectivities within our *lives in* society, centring discussions about causes and solutions for environmental emergencies on an individual level, such as personal choices related to consumption (e.g. greener products, recycling) and conservation activities (e.g. building bird houses, planting trees), without space for wider discussions about structural and political issues, and certainly without any support for collective action around these environmental issues and their associated injustices. Therefore, in the words of Santos (2009, p. 362) in his review of a Freirean perspective for science education, a socio-political turn in science education cannot be only about recognising the existence of the *social* in science, but also about engaging with its *political* angle in order to arrive to a truly emancipatory science education.

Within this scenario of a truly socio-political turn for science education, one that embraces the transformative nature of Freire’s work with marginalised groups, Tilsen proposes that science educators can learn from those groups that have been most involved in transforming our society through socio-political action: social movements. It is not surprising to see social movements more widely connected to discussions about socio-political action within education, especially under a Freirean perspective. Freire himself had a longstanding links with several Global South social movements throughout his life, such as the Landless Workers Movement in Brazil (Tarlau 2015) and the African Party for the Independence of Guinea-Bissau and Cape Verde (PAIGC), and his works are still very influential to current social movements across the Global South, as argued by Cassiani and von Linsingen in their work about East Timor in this special issue. But what I find particularly interesting in Tilsen’s article about the ACT UP (AIDS Coalition to Unleash Power) in the USA is the potential of Paulo Freire’s ideas around emancipation of marginalised communities to those, like me, working in the particular case of Global North contexts.

Several Global North societies, such in Tilsen’s article or in my own context as science educator in England, are both very culturally diverse and socially and culturally unequal, something that can be directly linked to complex socio-historical trajectories furthered by their own colonial and globalisation projects. So a question that emerges for me from Tilsen’s exploration of the case of a social movement in the USA through a Freirean lens is what science educators in the Global North can learn from Global South ideas and initiatives around socio-political transformation for science education, such as those inspired by Freire and associated decolonial and critical perspectives.

Although Freire is well known by academics in the Global North, the reality is that very few of his ideas actually find their ways to educational practices around here (Peters and Besley 2015) (although the same could be said for most mainstream educational practices in the Global South), clashing with decades of a neoliberal focus on standardisation, accountability and one-size-fits-all approaches as flagship of good education for social “inclusion” and “mobility” (Connell 2009; Giroux 2010). But, as recently argued by Stephen Ball and Jordi Collet-Sabé (2021), this promise of social inclusion and mobility within largely multicultural and unequal societies through this specific model of neoliberal Global North schooling is unachievable. Modern Global North education is epistemologically grounded on normalising and homogenising approaches, where diversity of ideas, ways of living and thinking are to be standardised into only one superficial way of, in Freirean terms, reading and sharing the world with others, leaving no space for reconstructing a different world that is not grounded on homogenisation and *othering* (Ball and Collet-Sabé 2021). In this scenario of an epistemological impossibility for science education in Global North schooling to become involved in socio-political transformation, Freire’s ideas—and the works of those actively attempting to bring them into the practice of science education, such as the authors in this special issue—are not only refreshing, but imperative to the lives of historically socially marginalised communities in these very culturally diverse parts of the world (Giroux 2010).

## Science teacher’s education and work for decolonial socio-political transformations

The teacher has to ask: what kind of politics am I doing in the classroom? That is, in favor of whom am I being a teacher? The teacher must also ask against whom I am educating; of course, the teacher must also be teaching in favor or something and against something. This ‘something’ is just the political project, the political profile of society, the political ‘dream’. (Shor and Freire 1987, p. 46)

Of common interest to Tilsen’s, Cassiani and von Linsingen, and Peñalosa et al.’s articles in this special issue is the role of teachers in a Freirean project for science education that supports a critical approach around issues of cultural diversity, decoloniality, and socio-political transformation. This is because, within a Freirean perspective, teachers play an important part, as argued by Freire across several of this works, such as *Teachers as Cultural Workers* (2018), and in his quote with Ira Shor above: teachers do not simply deliver knowledge to their students; they are engaged in political work “in favor or something and against something”.

Therefore, although unpacking this socio-political feature of teachers’ work in a comprehensive manner is beyond the scope of this article, it is important to remember here that science teaching, as with scientific endeavours, is a not neutral activity devoid of any norms, values or socio-political interests. Within this socio-political perspective on the teaching profession, the contexts from where the authors in this special issue write can, in different ways and to different extents, be positioned within larger global issues of pervasive neoliberal focus on educational performativity towards what Sharon Gewitz and Alan Cribb (2020) link to strategies of deliverology, datafication and metrification of education. As a result, an increased focus on large-scale data-driven educational performance of teachers and their students—as further explored by others like Stephen Ball (2003); Gert Biesta (2010); Raewyn Connell (2009) and Michael Domínguez (2019) has been pushing the nature of teaching across the world against Freirean ideals outlined in this special issue: from centred in interpersonal relationships where people (teachers, students and their communities) are recognised as socio-political actors, to centred in the delivery of a specific subset of content with a narrow set of accountabilities defined by external stakeholders and where teachers, students and communities are not recognised as socio-political sactors. That is, to what Freire (1972) famously linked to the *banking model of education*. Important aspects of educational practice connected to the kind of relational, critical and emancipatory education proposed by Freire, such as artistic, social and political work, have then been found too complicated and messy (and often politically undesirable) to be *delivered* and *measured* against these prevalent one-size-fits-all standards that characterise the *transactional teaching* that happens in a *banking model of education* (Gewitz and Cribb 2020). So, in this current transactional scenario of educational relationships, “teachers will have virtually no say over how the work of their students is going to be assessed and thus over what forms of knowledge matter” (Gewitz and Cribb 2020, p. 221).

Nevertheless, as highlighted by Cassiani and von Linsingen in this special issue, within a Freirean perspective that places the teaching profession as one centred in *relational* practice to challenge injustices and support emancipation, teachers cannot be positioned as *extensionists* (or *transactional* workers), but as facilitators of a dialogic work between themselves, students and communities; or, as asked for by Valladares (2021, p. 581), of a “dialogical and respectful exchange of diverse perspectives on the social and natural world”. And this way of thinking about teachers’ work is of special importance to those working with students and communities who have been historically marginalised from mainstream society; that is, those communities who have found themselves outside the standards that define what a good student should look like. As explored by teacher educators working closely with marginalised communities in this special issue and beyond, such as Keffrelyn Brown (2013) and Michael Domínguez (2019), any kind of critical culturally inclusive (science) education that aims at emancipating historically oppressed communities needs to counter processes of homogenisation of people’s identities and histories; and, for that to happen, teacher education and work need to be socioculturally critical and decolonial. This point was recently raised, for instance, by Brown (2013) and Domínguez (2019, p. 49) in the context of their work on initial teacher education committed to social justice and emancipation of marginalised communities in the USA:

*As a [teaching] profession, we have spent the better part of two, admittedly difficult, decades focused on ‘what works’ and trying to think of new ways to arrange novice’s interactions with diverse communities, only for research to tell us time and time again that the experiences historically marginalized youth have with schooling remain difficult, marginalizing, and even injurious. (…) [teachers’ work under an assimilative, one-size-fits-all logic is] a core of beliefs that validates Western ways of being, articulates exclusionary definitions of academic, personal, and social success, and privileges westernized knowledge and epistemic products, all while rendering everything else (the culture, social relations, phenotypes, ways of being in the world, knowledge, and epistemologies which emerge from the global south) as subaltern.*

Within the specific case of science education, we then find ourselves inquiring into the kinds of practices that are needed to support science teachers’ development and work towards the critical approach to issues of cultural diversity, decoloniality, and socio-political transformation within Freire’s project for an emancipatory education. Tilsen’s, Cassiani and von Linsingen’s, and Peñalosa et al.’s articles in this special issue all give us some important insights into how this type of work can be done: in the true spirit of Paulo Freire, not only students should be supported in developing their critical consciousness and engaging with socio-political transformation, but also science teachers themselves. And I would argue that this Freirean perspective on the profession might be even more imperative to the science teaching profession, where decades of a naïve perspective on the neutrality of science have led to an acute absence, in science teachers’ professional development, of an explicit engagement with science’s own norms, values or socio-political interests, as highlighted by Peñalosa et al. in this special issue and by others working in this area (e.g. Dunlop, Atkinson, Stubbs and Diepen 2021; Gandolfi 2022; Ideland 2018; Valladares 2021). In a world with increasingly complex injustices emerging from socio-scientific issues, such as environmental injustices and unequal access to healthcare (as exemplified by the current COVID-19 pandemic), my critical hope for science education certainly involves a deep rethinking of the science teaching profession.

And I also believe this *reimagining of the science teaching profession* to be an imperative not only to the communities in the Global South deeply impacted by centuries of marginalisation, such as the ones explored by Cassiani and von Linsingen and Peñaloza et al., but also to Global North societies that often led these socio-historical processes of oppression, such as the British Empire in my own professional context. The importance of the Freirean reflections developed throughout this special issue to those like me who work with teachers in the Global North is then twofold:

- To promote more humanising, anti-colonial and anti-oppressive practices among science teachers and their students in their own diverse and unequal communities;
- And to avoid perpetuating (neo)colonialism in/through science education via international development initiatives led by educators and researchers in the Global North, which are often grounded on assistentialist and homogenising perspectives on teacher education and work that do not recognise important scholarship and practices from Global South teachers, educators and researchers, as recently raised by Kofi Dompere (2020).

Thus, and in the spirit of transnational solidarity put forward across this special issue, the Freirean South-South cooperation on teacher education proposed by Cassiani and von Linsingen in their article could be an inspiration for us in the Global North interested in challenging our own struggles with standardisation and homogenisation of education that prevent us, like argued by Brown (2013) and Domínguez (2019), from properly engaging with issues of cultural diversity, decoloniality, and socio-political transformation within our own practices of science teacher education. Some initiatives in the Global North—as illustrated by several articles across this journal—have already been engaging with Freirean and decolonial perspectives around cultural inclusion in science education practices, and I hope this special issue will prompt others to rediscover Paulo Freire beyond his current instrumental presence in reference lists of academic publications.

## Final thoughts

At the start of this commentary article, I raised the question of “what can we learn from each other—as science educators—about the relevance of Freire’s ideas to the contemporary conceptualisation and practice of science education?”. As outlined across this special issue and, more generally, by the prevalence of Paulo Freire’s works in education and science education scholarship, several of his ideas seem to still be relevant to science educators across the world, even after more than 50 years since the publication of the *Pedagogy of the Oppressed* in a non-mainstream language within academia and based on the experiences of a thinker and educator in a deeply disadvantaged Global South community. As argued by several authors across this special issue and beyond, Freire’s proposals around critical consciousness, intercultural learning, socio-political action and transformation, teachers’ work, etc., can support our challenges to social injustices within and with science education, especially in this period of increasingly turbulent and complex environmental, social and economic issues across the world shaped by volatility, uncertainty, complexity and ambiguity (Valladares, 2021).

Nevertheless, while these Freirean ideas have been part of science education scholarship for decades, scholars such as Wilton Lodge (2021) have recently argued that their insertion into curricular and classroom practices around science have been less visible—if at all present—and I would say that this is specially the case in Global North education, from where Lodge also writes. As I explored above, the neoliberal focus on datafication and standardisation, which is so pervasive in how education (and teacher education) is framed and done in some Global North countries such as my own professional context, has made the work of science educators with Freirean ideas based on dialectic processes, critical consciousness, intercultural learning and problem-posing difficult, if not impossible. And as recently argued by Arthur Galamba and Brian Matthews (2021) and Lodge (2021) in this journal, this is specially the case in contexts where education has found itself not only within a neoliberal scenario, but also at the centre of right-wing repressive ideologies which attempt to prevent teachers and educators from engaging with socio-political discussions around, for instance, the systemic marginalisation of certain communities based on race, ethnicity, social class, gender, sexuality, etc. [see cases, for instance, in Brazil (https://www.bbc.co.uk/news/world-latin-america-48039435) and in England (https://theconversation.com/anticapitalism-wasnt-banned-in-english-classrooms-during-the-cold-war-why-is-it-now-147121)].

Since Freirean ideas are centred in socio-political discussions and actions that are in stark contrast to these growing repressive ideologies—and to the limits imposed by a neoliberal focus on datafication and standardisation—the challenge for us science teachers and educators in bringing them into curricular and classroom practices remains. But, in the spirit of one of Freire’s last publications—*Pedagogy of Hope*, in 1992—the inspirations and collaborations outlined by Tilsen, Cassiani and von Linsingen, and Peñaloza et al. in this special issue might give us hope of a couple of pathways that science education can tread towards more critically inclusive and transformational practices. And here we might not only “hope” as a noun (to wish, to aspire, to wait for, etc.), but “hope”—as proposed by Freire—as an action verb, leading to critical and collective transformative action in the world: *esperançar*.
